# Beyond the genome: the role of functional markers in contemporary plant breeding

**DOI:** 10.3389/fpls.2025.1637299

**Published:** 2025-08-05

**Authors:** Tae-Chun Park, Pransiskudura Chamara Silva, Thomas Lübberstedt, M. Paul Scott

**Affiliations:** ^1^ Department of Agronomy, Iowa State University, Ames, IA, United States; ^2^ Interdepartmental Plant Biology, Iowa State University, Ames, IA, United States; ^3^ Crop Development Center (CDC), University of Saskatchewan, Saskatoon, SK, Canada

**Keywords:** functional markers (FMs), random markers (RDMs), sequencing, genomic selection (GS), polymorphism

## Abstract

Functional markers (FMs) are derived from polymorphisms that confer phenotypic trait variation, making them powerful tools in plant breeding. Unlike random markers, for which trait associations are unknown, or at best established via linkage or quantitative trait locus (QTL) analysis, FMs are associated with causative polymorphisms, providing precise and reliable information for trait selection. Since the concept of FMs was first proposed in 2003, the emergence and adoption of technologies that were not available at the time have significantly advanced FM discovery and application by enhancing the ability to precisely identify causal variants underlying complex traits, which is a critical prerequisite for FM development. Novel technologies such as high-throughput sequencing, multi-omics, gene editing, and advanced computational tools have enabled the precise identification and functional validation of DNA polymorphisms associated with trait variation. FMs can be used in genomic selection (GS) and modern plant breeding programs by improving selection efficiency and accuracy. While FMs provide numerous benefits, challenges still remain regarding their stability and transferability, and innovative approaches to overcome these limitations are continually being explored. The role of FMs in plant breeding is expected to grow as functional annotation of genomes improves and technologies like genome editing become more accessible. These developments will enable breeders to effectively integrate FMs into breeding pipelines for accelerating genetic gains and addressing global agricultural challenges.

## 1 Introduction

Functional markers (FMs) are based on sequences that have been functionally characterized (Andersen and Lübberstedt, 2003; Salgotra and Neal Stewart, 2020). FMs can be developed from any type of DNA polymorphism. To qualify as FMs, polymorphisms between different alleles of genes must cause trait variation. FMs originate from quantitative or qualitative trait polymorphisms (QTPs), which include quantitative or qualitative trait nucleotides (QTNs), based on single nucleotide polymorphisms (SNPs), or indel polymorphisms (QTINDELs). Causes of allelic differences in trait expression include loss-of-function of mutations, changes in gene expression levels, or alterations in gene product structure. FMs are also known as perfect, precision, or diagnostic markers (Salgotra and Neal Stewart, 2020; Salgotra et al., 2014).

Random DNA markers (RDMs) report the state of polymorphisms in randomly selected positions in the genome. RDMs are distributed throughout the genome and are used to assess overall genetic diversity. They serve as tools in genetic mapping and diversity studies. These markers are relatively easy to construct and are effective for characterizing the genetic structure of diverse populations. However, RDMs lack a direct causal relationship with specific gene functions, which can limit their predictive power in marker-assisted selection (MAS). Due to recombination, the association between RDMs and target alleles weakens over successive generations. Despite these limitations, RDMs play a critical role in initial QTL mapping and genomic diversity analyses, providing essential baseline information for further genetic studies (Andersen and Lübberstedt, 2003; Bagge et al., 2007). While RDMs and FMs are indistinguishable from a technical perspective based on marker assays, their distinction lies in the association with traits (Andersen and Lübberstedt, 2003; Bagge et al., 2007). In applications such as MAS, where the goal is to efficiently transfer target traits into different genetic backgrounds, FMs provide a distinct advantage. These applications include marker-assisted backcrossing (MABC) (e.g., Frisch and Melchinger, 2001), F_2_ enrichment (Bonnett et al., 2005), and MAS (Lande and Thompson, 1990). The key advantage of FMs lies in their perfect association with target traits, which reduces the risk of false positives due to recombination and improves the accuracy of marker-trait associations (Guo et al., 2010). Therefore, FMs are preferable over RDMs when they are available for tracking specific genes in breeding programs (Hasan et al., 2021).

Although FMs are defined based on polymorphisms with a clearly demonstrated causal relationship to phenotypic variation, not all markers are functionally characterized from the outset. In particular, when the FM concept was first introduced in the early 2000s, limitations in genomic and functional genomics technologies often made it difficult to draw a clear distinction between RDMs and FMs. However, with technological advances, an increasing number of markers initially used as RDMs have since been experimentally validated and reclassified as FMs. For example, in maize, the *opaque2* (*o2*) gene was identified in 1964 as a key regulator of lysine content in the endosperm. It was not until the 1980s that simple sequence repeat (SSR) markers such as *phi057*, *umc1066*, and *phi112*, located within the *o2* gene, were developed by Pioneer and the University of Missouri. These markers were initially used as associative or linked markers to facilitate MAS in quality protein maize (QPM) breeding programs. However, later studies revealed that some of these polymorphisms are closely linked to, or even causative of, the trait by directly affecting gene function. Consequently, markers that were originally used as indirect indicators, or RMD, were later reclassified as gene-based or FMs after functional validation confirmed their biological effects on lysine content (Chand et al., 2024). In rice, a SNP marker within the *BADH2* gene, which was initially a simple association marker, became a FM when the gene was identified as the genetic determinant of 2-acetyl-1-pyrroline, the major compound responsible for aroma (Singh et al., 2022). In wheat, polymorphisms in the *Ppd-D1* gene were shown to play a major role in controlling flowering time, and markers targeting this gene are now widely used as FMs (Beales et al., 2007). These examples illustrate that the boundary between RDMs and FMs is not fixed, and marker classification can evolve with the emergence of new empirical evidence. This continuum is a critical aspect for understanding the historical development of FMs and evaluating their utility in practical crop breeding.

**Figure 1:**
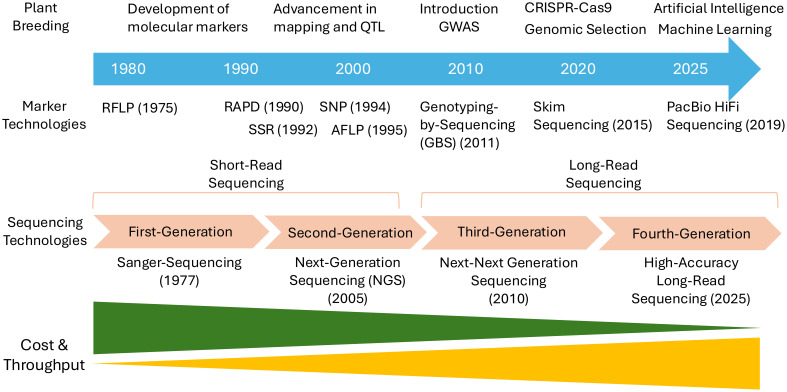
A timeline for the evolution of marker technologies and their integration into plant breeding from 1980s to the present.

The agricultural sector is currently facing a range of complex challenges, including climate change, emerging pests and diseases, soil degradation, and the urgent need to sustainably feed a growing global population (Hickey et al., 2019; Farooq et al., 2022). These pressures have significantly increased the demand for more precise, rapid, and efficient crop improvement strategies. In this context, FMs which directly target causal variants underlying phenotypic traits, offer high potential. Since the concept was first introduced in 2003 (Andersen and Lübberstedt, 2003), advancements in various technologies have greatly expanded the possibilities for FM development and application. High-throughput sequencing (Sun et al., 2022), genomic resources, and bioinformatics tools (Marsh et al., 2021) now enable the precise and efficient identification of causal variants for target traits (**Figure 1**). Furthermore, gene editing tools have provided experimental means to functionally validate candidate markers (Ahmar et al., 2020). Beyond traditional genome-wide marker effect-based GS, FMs are gaining attention as tools capable of directly capturing trait-associated variation, thus opening new possibilities for their integration into GS pipelines (Zhang et al., 2023). As such, FMs are no longer just molecular markers but are emerging as core components in breeding strategies aimed at dissecting and harnessing key genetic determinants of agronomic traits.

This paper provides a critical review of the progress in FM development and application in plant breeding since the publication of the FM concept in 2003. It includes, (i) the discovery and development of FMs using forward and reverse genetics, (ii) strategies for FM validation, (iii) the application of FMs in MAS and GS, (iv) recent expansions of FM applications including genome construction and gene editing-based breeding strategies, and (v) current challenges and future prospects for FM-based breeding.

## 2 Functional marker discovery and development

### 2.1 Forward genetics for gene and QTP identification

Forward genetics begins with an observable phenotype and aims to uncover the genes and genetic polymorphisms responsible for trait variation (Sahu et al., 2020). Over the past two decades, advances in mapping technologies, in particular the introduction of genome-wide association studies (GWAS), have greatly increased the precision of forward genetics. While QTL mapping locates larger genome regions affecting traits of interest, GWAS enables fine-scale detection of genetic variants. Map-based cloning is a strategy to confirm gene-trait associations rather than systematically identifying functional variants (Yu et al., 2021). This approach begins with a genetic mapping procedure such as QTL mapping, or other methods to localize genomic regions associated with the trait of interest (Shen et al., 2004). Once a target region has been identified, recombinant individuals are used to progressively narrow down the interval containing the causal gene. The population sizes required to identify causal polymorphisms at high resolution would need to be extremely large, likely in the tens of thousands or more. Thus, while map-based cloning is an established strategy to systematically clone genes for qualitative and quantitative traits, this approach is not feasible for QTP detection (Ganal et al., 2009; Watanabe et al., 2009).

Development of high-throughput and low-cost marker systems, such as Genotyping-by-Sequencing (GBS) were prerequisite for high-resolution GWAS (Thomson et al., 2022). These marker systems dramatically reduced the cost per sample, allowing for large populations to be analyzed. GWAS leverage high-density genotyping and populations with rapid linkage disequilibrium (LD) decay to fine-map candidate genes at high resolution. LD refers to the non-random association of alleles at different loci in a population. In plant breeding, LD is a crucial concept because it affects how genetic markers are associated with traits of interest (Flint-Garcia et al., 2003), and allows to efficiently pinpoint causal genetic variants. In QTL mapping populations, where LD decay is slow, large genomic regions spanning several mega-bases are identified, making it difficult to identify causal genes (Yan et al., 2009; Wallace et al., 2014). By exploiting low LD associations between specific polymorphisms and target traits in GWAS populations, candidate QTPs can be identified (Lu et al., 2010; Wallace et al., 2014). In maize, GWAS has revealed key genetic loci associated with agronomic traits. For example, SNPs associated with plant height have been linked to genes such as *Zm00001d018617* and *Zm00001d023659*, which are involved in gibberellin and auxin signaling pathways (Zhang et al., 2019). Similarly, husk number was associated with variation in the 3’ UTR of the ZMET2 gene, a DNA methyltransferase (Wang et al., 2025c). However, distinguishing true causal variants from those merely linked through LD remains a limitation. In maize, LD typically decays within 1–5 kb, depending on population structure and local genomic context (Flint-Garcia et al., 2005; Yan et al., 2011), underscoring the need for further functional validation of candidate QTPs.

**Figure 2:**
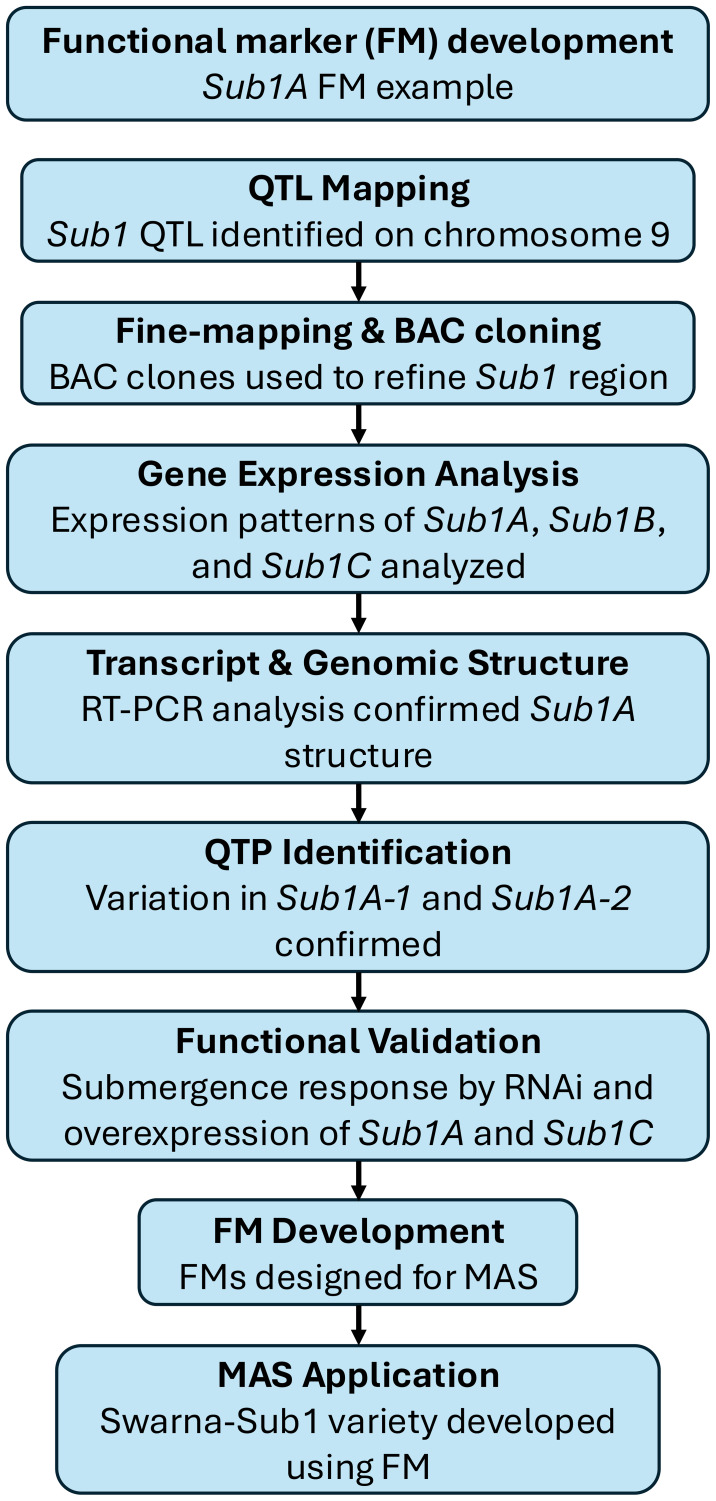
The example of functional marker (FM) development process for *Sub1A* in Rice (Xu et al., 2006). This diagram outlines the development of a FM for *Sub1A*, a key gene conferring submergence tolerance in rice. The process began with QTL mapping and fine-mapping using bacterial artificial chromosome (BAC) clones, leading to the identification of *Sub1A*. Gene expression analysis and reverse transcription-polymerase chain reaction (RT-PCR) confirmed its structure and differential expression. Quantitative trait polymorphism (QTP) identification revealed sequence variation between *Sub1A-1* (tolerant) and *Sub1A-2* (intolerant), followed by functional validation through RNA interference (RNAi) and overexpression studies. The FMs were then developed and applied in MAS, resulting in the *Swarna-Sub1* variety with improved flooding tolerance.

Some notable examples highlight the success of forward genetics in QTP discovery. For instance, the *Sub1A* gene in rice was identified by fine mapping of the *Sub1* QTL on chromosome 9 (**Figure 2**). Expression and functional analyses confirmed that *Sub1A-1*, a specific allele present in tolerant varieties, regulates submergence tolerance. A SNP in *Sub1A-1* was identified as a QTP associated with enhanced survival under flooding. FMs derived from *Sub1A-1* have been successfully implemented in MAS, leading to the development of submergence-tolerant rice varieties such as *Swarna-Sub1* (Xu et al., 2006). Similarly, the *MATRILINEAL* (*MTL*) gene in maize, encoding a patatin-like phospholipase, was identified as a key determinant of maternal haploid induction through GWAS and QTL mapping. Additional QTL, including a significant locus on chromosome 10 harboring a *Kokopelli* ortholog, were identified, confirming the polygenic nature of haploid induction rate (HIR) regulation. A frameshift mutation caused by a 4-bp insertion within *MTL* was validated as a QTP by demonstrating a strong association with increased HIR across diverse maize lines. FMs developed based on this QTP have been successfully used to enhance the efficiency of haploid inducer breeding (Trentin et al., 2023).

### 2.2 Reverse genetics for candidate gene and QTP identification

Reverse genetics enables researchers to directly verify the function of known genes by deliberately modifying them and observing the resulting phenotypic changes. In contrast to forward genetics, reverse genetics starts with a gene sequence and uses genomic sequencing, functional genomics, and gene editing technologies (Slade et al., 2005; Barkley and Wang, 2008) to assess its role in trait expression. By altering target genes through knockout, knockdown, or precise editing, researchers can evaluate the impact of specific allelic variants and establish causal links between gene function and phenotype.

In 2003, only Arabidopsis and rice had fully sequenced genomes (Yu et al., 2002; The Arabidopsis Genome Initiative, 2000). Today, most major crop species, including maize, have multiple high-quality genome assemblies available in public databases. For example, over 40 maize genomes are accessible in MaizeGDB (Portwood et al., 2019). This expansion enables researchers not only to work from a single reference genome, but also to explore allelic variation, presence/absence variation (PAV), and structural genome changes across diverse lines. Pan-genome resources capture genomic diversity beyond a single reference and enable development of broadly applicable markers. PAV offers particularly promising opportunities for designing highly selective markers that target lineage-specific or unique trait-associated genes (Hirsch et al., 2014). These advances have deepened our understanding of core and orphan genes and made it increasingly feasible to link polymorphisms with biological function (Gao et al., 2019). Nevertheless, while access to extensive genome sequence resources greatly enhances candidate gene discovery, it does not replace the necessity of validating QTPs through experimental studies.

Candidate genes identified by GWAS often serve as starting point for reverse genetics studies. Once loci associated with traits are discovered, targeted mutagenesis or gene editing can be applied to validate gene functions or those of specific polymorphisms. Mutagenesis arises from spontaneous errors during DNA replication and can be artificially induced using a variety of methods, such as chemical mutagenesis (e.g., ethyl methanesulfonate, EMS), physical treatments (e.g., ultraviolet radiation, UV or gamma irradiation), and biological approaches such as transposon insertions and targeted gene-editing technologies like CRISPR/Cas9 (Miao et al., 2013; Udage, 2021; Chen et al., 2023a). Mutagenesis enables validation of the function of specific polymorphisms. By deliberately introducing mutations, scientists can observe the resulting changes in phenotype, thereby separate causal from linked variants to elucidate gene function (Oladosu et al., 2016).

Transposons are mobile genetic elements that are integrated into the genome. They can disrupt gene sequences and regulatory regions. Transposon insertion and excision events can lead to loss-of-function or gain-of-function mutations. Insertion events caused by transposons can lead to loss-of-function or gain-of-function mutations. Transposon tagging has advanced significantly through the integration of NGS, enabling high-throughput identification of insertion sites. Large-scale mutant libraries with mapped or sequenced insertions have been developed in model and crop species, supporting genome-wide reverse genetics studies (Cain et al., 2020; Johnson et al., 2021). One example of using transposons for reverse genetic identification in maize is the Ac/Ds system applied to the *teosinte branched1* (*tb1*) gene. In this approach, Ds transposons were mobilized in the presence of the autonomous Ac element and inserted randomly into the genome. Through high-throughput sequencing of Ds insertion sites, researchers identified a Ds-tagged allele disrupting the tb1 gene, which was previously known to control plant architecture. The tagged mutants displayed altered tillering phenotypes, validating the gene’s function. This insertional mutant population, with sequenced Ds locations, allowed rapid identification of candidate genes and their associated stocks, providing a valuable resource for reverse genetics studies in maize (Studer et al., 2011).

Targeting Induced Local Lesions In Genomics (TILLING) uses chemical or physical mutagens to generate genetic variation and discover beneficial or novel alleles (Barkley and Wang, 2008). TILLING can be applied as reverse-genetics tool in any plant species. It facilitates the speedy and inexpensive generation of induced point mutations (G/C to A/T transition distributed randomly in the genomes) as well as the study of the functions of specific genes in mutants (McCallum et al., 2000). A public reverse genetics TILLING platform is available at the UC Davis Genome Center (https://genomecenter.ucdavis.edu/), providing EMS-induced mutant populations and sequencing-based screening for rice, wheat, Arabidopsis, and tomato (Comai and Henikoff, 2006; Studer et al., 2011). Despite the randomness of the induced mutations, systematically screening large mutant populations enables to detect mutations in genes of interest (Gilchrist and Haughn, 2005). In wheat, for example, TILLING has enabled the identification of novel alleles in functionally relevant genes such as *Wx-A1*, *Wx-D1*, and *Ppd-D1*, which are associated with starch composition and flowering time, respectively (Dong et al., 2009; Chen et al., 2012). In contrast, EcoTILLING uncovers natural genetic variation in specific genes, eliminating the need for artificial mutagenesis (Garvin and Gharett, 2007; Backes, 2013). SequeTILLING has been proposed as an extension of TILLING with the help of NGS techniques (Weil and Monde, 2009; Backes, 2013). One disadvantage of TILLING is the presence of background mutations that can affect the phenotype and, hence, impede gene function analysis (Szurman-Zubrzycka et al., 2023). Backcrossing may be needed, which is time-consuming. TILLING has inherent limitations. To induce mutations at every nucleotide position within a target gene, an extremely large mutant population would be required which is not feasible, both experimentally and logistically. Thus, the probability of accurately targeting and identifying a specific QTP by TILLING is low. While TILLING is useful for identifying candidate genes, it has limited utility for the precise validation of specific QTPs (Tsai et al., 2011).

**Figure 3:**
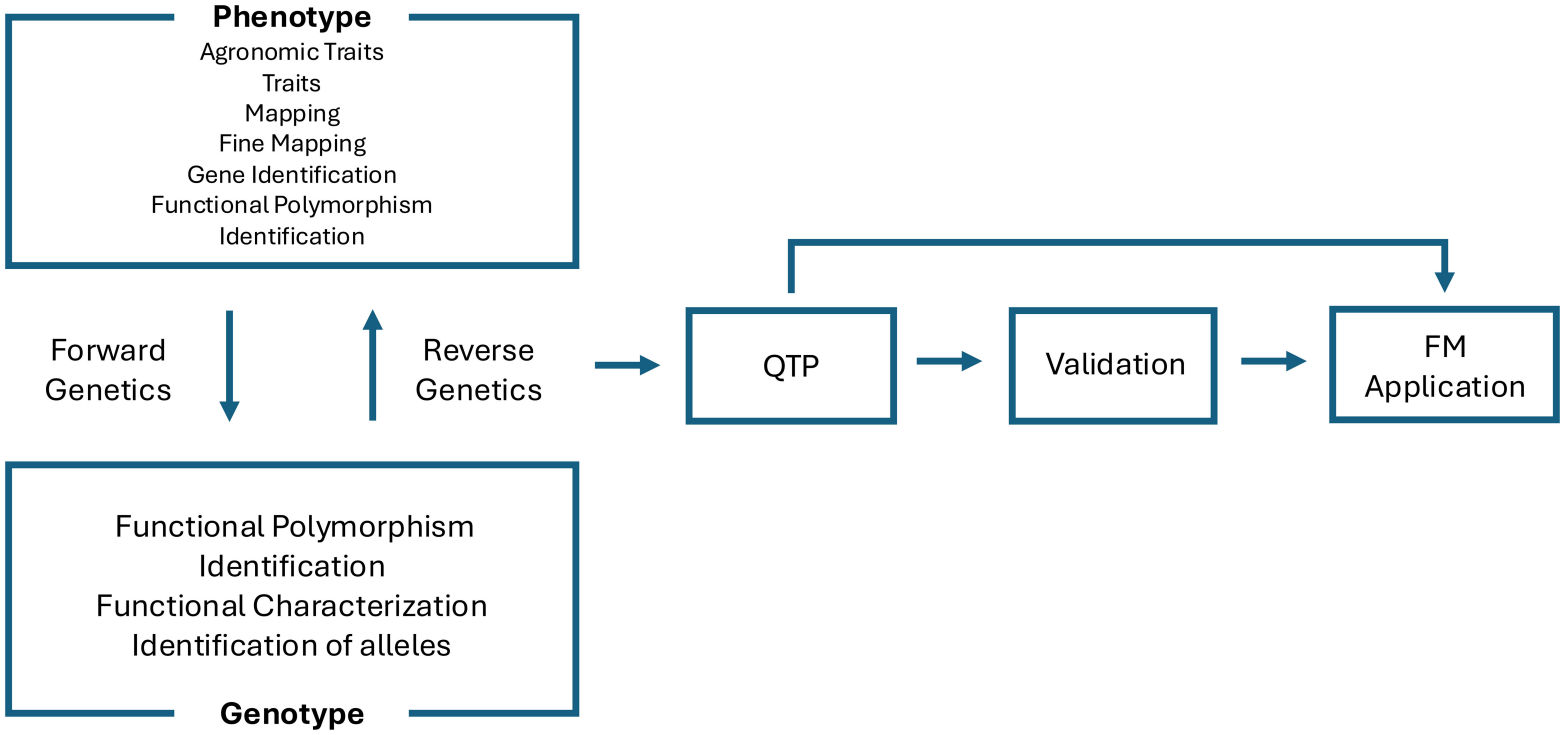
A schematic overview illustrating how forward and reverse genetic approaches link various phenotypic traits to genotypic information. Quantitative trait polymorphism (QTP) identification and validation concludes in Functional Marker (FM) application.

Finally, the increasing application of artificial intelligence (AI) and machine learning (ML) models has expanded the toolkit for QTP validation. Deep learning models trained on genomic, transcriptomic, and epigenomic datasets have been used to predict regulatory elements and prioritize functional variants. Notably, AI-guided prioritization of candidate genes has been applied in maize and soybean to narrow down GWAS signals and select variants for functional assays (Washburn et al., 2019; Zhang et al., 2023). As such, AI-assisted validation strategies are expected to play an increasingly important role in bridging large-scale association data with FM development (Washburn et al., 2019; Yoosefzadeh-Najafabadi et al., 2022) (**Figure 3**).

## 3 Validation of FMs

The primary goal of validation is to firmly establish a causal relationship between a specific genetic variant and trait expression. Validation is necessary when candidate QTPs have been identified through statistical associations. In some cases, validation methods not only confirm gene function but lead to discovery of new genes or QTPs. For example, gene editing approaches such as CRISPR/Cas9 can be used to create targeted mutations, revealing novel insights into gene function beyond initial candidate predictions. Thus, validation serves both as a confirmation tool and a discovery mechanism (Gaj et al., 2013; Arora and Narula, 2017). Recent advances in gene editing over the past decade have enabled precise, targeted mutagenesis, providing a more efficient and broadly applicable approach.

Gene editing begins with careful selection of a target gene and the design of specific mutations based on predicted functional domains or regulatory elements thought to be critical for gene activity. Subsequently, isogenic mutant and wildtype genotypes are subjected to comprehensive phenotypic analyses. These comparative studies reveal, how the specific alteration impacts the biological processes and trait expression, thereby establishing a direct link between the engineered mutation and its phenotypic effect. Moreover, by systematically correlating these changes with gene function, this approach not only validates candidate genes but also aids in validation of QTPs (Gilchrist and Haughn, 2010; Saika et al., 2011).

CRISPR/Cas9 targets specific genomic sites by generating double-strand breaks (DSBs) at user-defined sequences using a single guide RNA (sgRNA) that directs the Cas9 nuclease to its target (Ferreira and Choupina, 2022). Once the DSB is induced, the cell predominantly employs the non-homologous end joining (NHEJ) repair pathway. NHEJ is one of the primary pathways for repairing DSBs in DNA, playing a crucial role in cellular DNA damage repair mechanisms (Chang et al., 2017). NHEJ functions by directly ligating the broken DNA ends, often introducing small Indels in the process. Due to its error-prone nature, NHEJ is more useful for intentionally disrupting genes to assess whether a gene is causally linked to loss-of-function phenotypes, rather than for validation of specific QTPs. In contrast, targeted genome editing tools such as TALENs CRISPR-Cas with homology-directed repair (HDR), or base editing offer higher precision and are more suitable for validating QTPs (Zhang et al., 2017; Chen et al., 2019; Ferreira and Choupina, 2022). However, HDR efficiency is generally low in plant cells due to the strong preference for NHEJ over HDR for repairing double-strand breaks. This limitation is particularly evident in somatic tissues, making the practical application of HDR in plant systems are more challenging. To overcome these constraints, various strategies have been developed, including the use of single-stranded oligodeoxynucleotides (ssODNs) as repair templates (Schubert et al., 2021), synchronization of the cell cycle to favor HDR activity (Lin et al., 2014), and the application of NHEJ inhibitors (Yang et al., 2020). Despite its lower efficiency, HDR remains a critical tool for precise genome editing, especially in validating candidate FMs.

Base editing is an innovative CRISPR-derived technology that enables the precise conversion of one nucleotide to another without inducing DSBs (Gaj et al., 2013; Kantor et al., 2020; Xu et al., 2021). This method employs a fusion of a catalytically impaired Cas9 (or Cas9 nickase) with a deaminase enzyme, allowing targeted C-to-T or A-to-G substitutions. Its precision is particularly beneficial for creating SNPs (Salgotra and Neal Stewart, 2020). By avoiding DSBs, base editing minimizes the risk of undesired insertions or deletions and off-target effects, enhancing its reliability for QTP validation. Recent studies in rice have demonstrated the potential of base editing for multiplexed nucleotide modifications, making it a valuable tool for precise allele correction and crop improvement (Zong et al., 2017; Kantor et al., 2020; Xu et al., 2021). Prime editing represents a next-generation approach that extends the capabilities of precise genome modifications by allowing all 12 types of base substitutions, as well as small insertions and deletions, without the need for DSBs or donor DNA templates (Anzalone et al., 2019; Thomson et al., 2022). This technique utilizes a fusion protein combining a Cas9 nickase with a reverse transcriptase, guided by a prime editing guide RNA (pegRNA) that encodes the desired edit. Although still emerging, prime editing holds significant promise for accurately delivering beneficial natural mutations (Kantor et al., 2020; Thomson et al., 2022).

While gene editing enables precise genome modifications at the single-nucleotide level, increasing attention has been directed toward technologies capable of generating or validating larger Indels and structural variations at the chromosomal scale. Notably, CRISPR-Cas9 was used to induce a pericentric inversion spanning 75.5 Mb on chromosome 2 in maize, demonstrating the feasibility of engineering large-scale genomic rearrangements previously unattainable with conventional editing tools (Schwartz et al., 2020). Some studies have further extended this capacity using Cas3-based systems, achieving targeted genomic deletions exceeding 200 kb (Csörgő et al., 2020). Together, these examples highlight the growing potential of advanced genome editing platforms for functional gene analysis and QTP validation, expanding the scope of FM development beyond simple SNP-level variation.

The recently developed non-editing approach Fast Identification of Nucleotide variants by droplet DigITal PCR (FIND-IT) offers a promising alternative for QTP validation. FIND-IT combines large-scale mutagenesis with systematic large-scale pooled genotyping to identify rare mutants, and subsequent genomic and phenomics characterization to validate candidate QTPs. This approach is particularly valuable in species where transformation and editing remain technically challenging or time-consuming (Knudsen et al., 2022). FIND-IT enables high-throughput mutation-to-phenotype mapping through pooled genotyping and droplet digital PCR (ddPCR), offering greater efficiency than traditional TILLING without requiring transgenesis or targeted editing (Huang et al., 2023). The FIND-IT technology was used to screen a mutant library consisting of approximately 500,000 barley individuals. As a result, more than 125 functional alleles were successfully identified. This demonstrates that combining large-scale EMS-induced mutant populations with high-throughput phenotyping is an effective strategy for validating.

TILLING was employed to generate powdery mildew-resistant hexaploidy bread wheat by targeting *TaMlo* genes, orthologues of the barley *Mlo* gene, which confers durable resistance to *Blumeria graminis f.* sp. *Tritici* (*Bgt)*. By high-resolution melting (HRM) analysis, 16 missense mutations were identified in *TaMlo-A1*, *TaMlo-B1*, and *TaMlo-D1*, with functional validation in a barley transient expression assay confirming that specific mutations conferred reduced *Mlo* function and increased resistance. Homozygous triple mutants (*tamlo-aabbdd*) exhibited strong resistance to *Bgt* without pleiotropic effects such as early leaf senescence. This study demonstrates the effectiveness of non-transgenic gene editing approaches in improving disease resistance in wheat (Acevedo-Garcia et al., 2017). CRISPR-based cytosine base editing (CBE) was used to fine-tune amylose content in rice by precisely modifying the *Wx* gene, which encodes granule-bound starch synthase I (GBSSI), a key enzyme in amylose biosynthesis. Three sgRNAs were designed to introduce specific base substitutions in the N-terminal domain of *Wx*, generating rice lines with amylose levels ranging from 1.4% to 11.9%, enabling precise control over grain quality. Genotypic analysis confirmed stable inheritance of the mutations, with no detectable off-target effects. The study highlights how CRISPR base editing can be leveraged to modify starch composition, improving eating and cooking quality in rice (Xu et al., 2021). CRISPR-Cas9 genome editing was used to modify the *ARGOS8* gene in maize, improving drought tolerance and grain yield. The *ARGOS8* promoter was replaced with the *GOS2* promoter or inserted into the 5’-UTR, leading to increased expression. qRT-PCR analysis confirmed enhanced transcript levels, validating the modification. Field trials demonstrated a 5-bushel per acre yield increase under drought conditions without yield loss in well-watered environments. This study highlights the potential of precise genome editing for improving complex agronomic traits in maize (Shi et al., 2017). In soybean, CRISPR-Cas9-mediated editing of the *FAD2–2* gene significantly increased oleic acid content by disrupting the microsomal omega-6 desaturase enzyme, which converts oleic acid to linoleic acid. The targeted mutations, introduced using a single-guide RNA (sgRNA), resulted in up to a 65.9% oleic acid content in mutant lines, as confirmed by Near-Infrared Spectroscopy (NIR) analysis. No off-target effects were detected, demonstrating the specificity and efficiency of CRISPR-based genome editing in improving soybean oil quality (Al Amin et al., 2019) (**Table 1**).

**Table 1:** Comparison of three functional marker (FM) validation methods addressed in this paper.

	TILLING (Targeting Induced Local Lesions IN Genomes)	CRISPR-based validation	FIND-IT (Fast Identification of Nucleotide variants by droplet DigITal PCR)
Mutation Source	Induced mutations	Targeted gene editing	Natural or induced variants
Specificity	Low (random mutations)	Very high (precise targeting)	Moderate to high (known variant-based)
Detection Method	Mismatch cleavage, sequencing	Genotyping of edited lines (e.g., sequencing)	Droplet digital PCR (ddPCR)
Speed & Efficiency	Slow, labor-intensive	Moderate to high	Fast, high-throughput
Functional Confirmation	Phenotypic analysis of mutants	Direct gene function validation	Screening rare beneficial alleles
Typical Application	Loss of function screening	QTP validation	Mining useful variants in large pools

The table summarized key features of Targeting Induced Local Lesions IN Genomes (TILLING), CRISPR-based validation, and Fast Identification of Nucleotide variants by droplet DigITal PCR (FINT-IT) in terms of detection mechanism, resolution, speed, and efficiency.

## 4 Application of FMs in plant breeding

### 4.1 FMs for marker-assisted backcrossing and selection

The following examples were selected to illustrate the diverse applications of FMs across major crops, including wheat, rice, and maize. These cases represent both widely adopted and more specific uses of FMs in breeding programs, ranging from disease resistance to grain quality traits. In total, six representative cases are highlighted, demonstrating how functionally validated polymorphisms have been successfully integrated into MAS strategies. FMs should be based on functionally validated QTPs, where they are derived from two homozygous genotypes with identical genetic backgrounds but differing QTP alleles, showing clear phenotypic differences for the target trait. Consequently, fully validated QTPs in plants remain still rare.

MAS has been instrumental in addressing simple and oligogenic inherited traits, particularly through methods such as MABC, gene pyramiding, and F_2_ enrichment (Cobb et al., 2019). These approaches have been utilized for more than 20 years to efficiently tackle challenges like linkage drag, where undesirable genes are inherited along with target traits. Progress in marker technologies has significantly improved the efficiency and precision of these methods, enabling breeders to better address these challenges in complex breeding scenarios. In MABC, FMs help track the introgression of beneficial traits from donor parents into recipient lines by identifying alleles associated with those traits. This is particularly important when transferring beneficial traits such as disease resistance or stress tolerance. By targeting specific functional loci, FMs ensure that the desired traits are retained. In gene pyramiding, FMs enable the simultaneous selection of multiple favorable alleles from different parents, combining them into a single genotype with improved performance. This approach is critical for developing crop varieties with stacked traits, such as combined resistance to multiple pathogens. For F_2_ enrichment, FMs allow for the early identification of individuals carrying favorable alleles in segregating populations, streamlining the breeding process by focusing resources on the most promising candidates (Collard and Mackill, 2008; Salgotra and Neal Stewart, 2020). These precise applications of FMs significantly enhance the effectiveness of MAS for simple and oligogenic traits (Kumar et al., 2019).

The *Lr34/Yr18/Pm38* locus provides durable, non-race-specific resistance to multiple fungal pathogens, including leaf rust (*Puccinia triticina*), stripe rust (*Puccinia striiformis*), and powdery mildew (*Blumeria graminis*). This resistance is controlled by a single gene encoding an ATP-binding cassette (ABC) transporter, with resistant and susceptible alleles differing by only three sequence polymorphisms (Lagudah et al., 2009). The gene originates from certain wheat landraces and has been introgressed into modern wheat cultivars through MAS. To facilitate its use in breeding, FMs (*cssfr1–cssfr6*) were developed, enabling precise selection of resistant genotypes. These markers were validated in a diverse set of wheat cultivars, improving breeding efficiency by allowing early-generation screening for durable resistance without the need for pathogen exposure. Similarly, the *Yr36* (*WKS1*) gene, which confers non-race-specific stripe rust resistance at elevated temperatures, was originally identified in wild wheat but was largely absent in modern bread and pasta wheat varieties due to domestication bottlenecks (Fu et al., 2009). FMs for *Yr36* enabled its targeted reintroduction into elite wheat germplasm via MAS, ensuring the incorporation of resistance without disrupting agronomically favorable traits (Fu et al., 2009; Lagudah et al., 2009). Amylose content (AC) is a key determinant of rice grain cooking and eating quality, with the *Waxy* (*Wx*) gene playing a major role in its regulation. The *Wx* gene encodes *granule-bound starch synthase I* (*GBSSI*), an enzyme critical for amylose biosynthesis. A SNP in exon 6 (*Ex6A/C*), which results in an amino acid substitution from serine to tyrosine, is associated with intermediate amylose content (*Wx-in*). To facilitate selection for this trait in breeding programs, a FM was developed using polymerase chain reaction with confronting two-pair primers (PCR-CTPP) in a single-tube assay. This marker allows for efficient and cost-effective genotyping of *Wx-in* alleles. The marker was validated in a Chinese mini core collection (*Oryza sativa L.*) and a breeding population, demonstrating high specificity and applicability. By enabling precise selection of genotypes with intermediate amylose content, this marker streamlines the development of rice varieties tailored for consumer preferences in different markets. Its use in breeding programs ensures accurate trait selection without reliance on labor-intensive biochemical assays (Zhou et al., 2018). Moreover, *Fusarium head blight1* (*FHB1*) is a major fungal disease affecting wheat and barley, causing significant yield losses and grain contamination. Fhb1 is the most widely recognized QTL for *FHB1* resistance, playing a crucial role in reducing disease severity. FMs were developed based on a critical sequence deletion in the *TaHRC* gene within the *Fhb1* region and were validated in a global wheat collection. Comparative genomic analysis between near-isogenic lines (NILs) with contrasting *Fhb1* alleles enabled the identification of two diagnostic markers. Haplotype and sequence analyses across multiple genetic diversity panels confirmed their effectiveness, demonstrating higher selection accuracy than previously used markers. These markers provide a precise and efficient tool for MAS in wheat breeding, facilitating the development of *FHB1*-resistant cultivars and improving disease management strategies (Su et al., 2018).

The development of maize hybrids with improved tolerance to drought and low nitrogen, along with higher provitamin A (PVA) levels in sub-Saharan Africa, can be facilitated by refining and confirming the function of PVA-associated genes in regionally adapted inbred lines (Obeng-Bio et al., 2020). This study aimed to evaluate drought and low-N tolerance and PVA concentrations in early-maturing PVA-quality protein maize (QPM) inbred lines and to identify inbred lines carrying the *crtRB1* and *LcyE* genes as potential sources of favorable PVA alleles. Seventy early-maturing PVA-QPM inbred lines were evaluated under drought, low-N, and optimal conditions in Nigeria over two years. PVA levels were quantified, and allele-specific PCR markers were used to detect the presence of PVA-associated genes. The inbred lines exhibited moderate variation in PVA content; however, the TZEIORQ 55 line demonstrated both high PVA concentration and tolerance to drought and low-N stress. Analysis using the *crtRB1-3*′TE primer and the KASP SNP marker (snpZM0015) consistently identified nine inbred lines, including TZEIORQ 55, harboring the favorable *crtRB1* allele. These inbred lines represent valuable genetic resources for PVA biofortification in maize breeding programs (Obeng-Bio et al., 2020).

### 4.2 Marker-assisted approaches with FMs including genomic selection

While MAS has been effective for simple or oligogenic traits, it has proven less effective for quantitative traits, which involve polygenic inheritance and complex genetic interactions. Moreover, MAS is advantageous for selecting simple traits, but for complex traits, GS, which is based on information throughout the genome, is more effective. Therefore, efforts to integrate FMs into GS models have emerged (Bhat et al., 2021; Sivabharathi et al., 2024). By decoupling selection from the need for extensive phenotyping, GS improves breeding efficiency and scalability for modern breeding programs. Although GS does not require FMs, recent advancements have shown that incorporating fixed effects for known genes can further improve prediction accuracies. In particular, GS models incorporating known genes can significantly enhance the prediction accuracy of complex traits (Jeong et al., 2020). For example, integrating FMs associated with specific disease resistance genes into GS models has improved resistance prediction across various crops (Zhang et al., 2021). This approach combines the strengths of traditional MAS with advanced genomic prediction methodologies. Spindel et al. (2016) showed that FMs enhance GS accuracy by incorporating significant markers identified through GWAS as fixed effects in GS models. This integration provides population-specific insights into genetic architecture, improving the reliability of genome estimated breeding value (GEBV) predictions and supporting efficient breeding designs. Ultimately, FMs contributes to maximizing genetic gains while maintaining genetic diversity. Chen et al. (2025), moreover, proposed a model multi-trait ridge regression BLUP (rrBLUP) and *de novo* GWAS to improve genomic prediction accuracy for agronomic traits in maize. FMs identified through *de novo* GWAS were incorporated as fixed effects, significantly enhancing prediction accuracy for low-heritability traits. The multi-trait models further leveraged genetic correlations among traits to improve performance. Bayesian and multi-trait models outperformed rrBLUP for certain traits, particularly when known FMs were included as fixed effects. This suggests that integrating FMs into GS models can enhance prediction accuracy, thereby improving the efficiency of selection in breeding programs.

GS models that incorporate known genes have significantly advanced the field of plant breeding by enhancing the accuracy and efficiency of trait prediction. These models could leverage FMs that are directly associated with specific genes known to influence desirable traits, such as disease resistance, yield, and abiotic stress tolerance. By integrating these known genes into GS models, breeders can more precisely predict the performance of breeding lines and make more informed selection decisions (Jeong et al., 2020; Salgotra and Neal Stewart, 2020; Chen et al., 2023c). For instance, in maize breeding, integrating FMs associated with disease resistance genes such as the *Ht1* gene for northern corn leaf blight resistance has improved the accuracy of resistance prediction (Technow et al., 2013; Hurni et al., 2015). This integration allows breeders to not only select for high-yielding varieties but also ensure that these varieties possess robust disease resistance. Similarly, in wheat, incorporating markers linked to the *Rht-B1* and *Rht-D1* genes, which control plant height and lodging resistance, into GS models has facilitated the development of semi-dwarf varieties that are less lodging and higher yield potential (Zanke et al., 2014). Moreover, the use of known genes in GS models can help address the “large *p*, small *n*” problem, where the number of markers (p) greatly exceeds the number of phenotypic records (n). By focusing on a subset of markers with known effects, the model complexity is reduced, leading to more stable and reliable predictions (Robertsen et al., 2019). Additionally, the incorporation of these known FMs can improve the transferability of GS models across different breeding populations and environments, as the effects of these markers are more consistent and well-understood. This approach not only enhances the prediction accuracy but also accelerates the breeding cycle by enabling earlier selection of superior genotypes (Cerrudo et al., 2018). Overall, the integration of known genes into GS models enhances the efficiency and effectiveness of plant breeding programs.

### 4.3 Expansion of FM applications

FMs are not limited to their traditional use such as MAS and backcrossing but also have a much broader scope of application in plant breeding, particularly with the advancement of new technologies. In modern plant breeding programs, FMs can enhance the utilization in a way of innovative approaches such as haploid inducer-edit (HI-Edit), genome construction, and promotion of alleles by genome editing (PAGE). HI-Edit is an approach that leverages functionally validated QTPs to directly introduce specific alleles into target genotypes through precise gene editing. Unlike traditional MAS, which relies on marker-based selection, HI-edit directly modifies the genome at the QTP sites, enabling rapid validation and application of functional polymorphisms (Delzer et al., 2024). For instance, by directly introducing a beneficial QTP allele into an elite line, researchers can efficiently test and confirm its phenotypic effect, providing a more accurate understanding of gene-trait relationships. PAGE is an advanced method that directly increases the frequency of favorable alleles through genome editing, significantly enhancing genetic gain compared to traditional GS (Hickey et al., 2016). By directly targeting specific genes or QTPs, PAGE provides a unique opportunity to leverage FMs more efficiently. Based on this background, a strategy can be proposed that integrates PAGE and FMs for application in plant breeding. Specifically, functionally validated QTPs identified through FMs can be used to detect favorable alleles, which can then be directly edited using PAGE to accelerate genetic improvement. This approach is expected to be particularly useful for crops where precise enhancement of complex traits is required. Genome construction in plant breeding is a strategy that involves designing, assembling, and optimizing genetic configurations within a plant genome to achieve desired traits (Varshney et al., 2021). Unlike traditional breeding methods, which rely on random genetic recombination, genome construction is a targeted approach that systematically combines beneficial genes and QTPs to create superior plant varieties. FMs enhance genome construction by enabling precise selection of functionally validated QTPs, ensuring that only beneficial alleles are included in the final genome design. This accelerates trait improvement, minimizes genetic drag, and increases the reliability of selected traits.

## 5 Challenges and future perspectives

### 5.1 Limitations in FM development and application

FMs are increasingly valuable tools in plant breeding due to their ability to directly associate genetic variation with phenotypic traits, offering precision in selecting desirable traits and accelerating the breeding process (Kage et al., 2016; Salgotra and Neal Stewart, 2020). However, the application of FMs is not without limitations. Key challenges include genetic background differences, environmental influences (E), and genotype-by-environment (GxE) interactions, all of which can affect the stability and transferability of FMs. Additionally, epistasis, which manifests as genotype-by-genotype (GxG) interactions, can complicate the predictability of FMs by altering the effects of individual loci depending on the genetic background. Furthermore, incomplete penetrance can result in variability in trait expression even when the causal allele is present. These factors necessitate thorough validation and careful consideration in breeding schemes. In some cases, a single gene may contain redundant multiple QTPs that each contribute to the same phenotype. Traditional FM approaches often focus on a single SNP or specific QTP to predict a trait, potentially overlooking other polymorphisms that also influence the phenotype. This redundancy means that the information provided by an individual QTP may be duplicated by adjacent QTPs, and a marker based only on one may fail to capture the cumulative effect of the entire gene. In contrast, haplotype-based approaches combine multiple SNPs within a gene to represent the optimal haplotype that reflects all functional variations. Such an approach accounts for both additive and epistatic interactions among QTPs, thereby offering a more comprehensive view of the genotype–phenotype relationship. Integrating this strategy into FM development enhances the precision and stability of MAS, ensuring that the selected alleles truly represent the optimal genetic configuration. Recent studies advocate for the use of haplotype-based methods to overcome the limitations inherent in single-QTP markers (Bhat et al., 2021; Sivabharathi et al., 2024). To further enhance the utility of haplotype-based FMs, recent advances in computational tools such as Beagle and SHAPEIT4 have enabled accurate haplotype phasing and imputation, even in complex genomic regions (Browning and Browning, 2007; Delaneau et al., 2019). These tools facilitate the identification of functionally relevant haplotypes by resolving linkage patterns and structural variations. Integration with transcriptomic and epigenomic data, including expression haplotypes (eHaps) and expression quantitative trait locus (eQTL), informed haplotypes, improves the biological resolution of genotype, phenotype associations (Chien et al., 2023). In rice and wheat, for example, haplotype analyses at key loci such as *GW3*, *GW5*, and *FHB1* have enabled the selection of elite alleles for grain shape, and disease resistance (Radecka-Janusik et al., 2022; Xia et al., 2025). Moreover, incorporating haplotype-based markers into GS models has shown improved prediction accuracy, especially for traits influenced by multiple interacting loci (Alemu et al., 2023). This makes haplotype-based FM development a promising strategy for precision breeding under complex trait architectures.

One obstacle is the presence of repetitive sequences in plant genomes, which can complicate primer design and limit the specificity of marker assays. This is particularly problematic in large, complex, or polyploid genomes such as those of wheat or sugarcane, where homoeologous and paralogous sequences share high similarity across subgenomes or gene families. Such redundancy makes it difficult to design markers that uniquely target a single locus, increasing the risk of cross-amplification or false positives in genotyping assays (Claros et al., 2012). Another significant limitation is gene functional redundancy. In many plant species, important agronomic traits are controlled not by single genes but by gene families with overlapping or compensatory functions. When one gene is mutated, other family members may mask the phenotypic effect, thereby reducing the likelihood of detecting a clear genotype–phenotype relationship. This makes it challenging to identify causative polymorphisms suitable for FM development, especially in cases where loss-of-function alleles do not lead to observable trait variation (Peng, 2019). To mitigate such challenges in practical breeding, several crop-specific strategies have been implemented. For instance, in rice, multi-environment QTL mapping has been used to identify stable loci for drought tolerance, which are less affected by G×E interactions (Dixit et al., 2014). In maize, environment-specific genomic prediction models incorporating FM information have improved trait predictability under varying stress conditions (Rogers and Holland, 2022). These examples demonstrate that integrating FMs with tailored breeding strategies can enhance marker robustness and increase their utility across diverse genetic and environmental contexts.

Genetic background effects influence the stability of FMs, often preventing a marker from performing consistently across diverse genetic backgrounds. These effects arise due to epistasis, where interactions between different loci modify the expression of a target allele. For instance, a FM designed based on a specific genetic variation may work effectively in one genetic background but fail to produce the expected phenotype in another due to differences in interactions with other background genes. This occurs because the phenotypic effect of a given allele is not solely determined by its presence but also by how it interacts with other alleles in the genome. If these interacting loci vary between backgrounds, the expected effect of the FM may not be observed. These interactions can amplify or suppress the marker’s effects, leading to phenotypic variability even among individuals carrying the same marker. To ensure the stability of FMs, thorough validation across diverse genetic backgrounds and environments is necessary, along with strategies to minimize the influence of background effects (Guo et al., 2010; Salgotra and Neal Stewart, 2020). The environment (E) plays a crucial role in the stability and effectiveness of FMs. Environmental factors regulate gene expression, altering the phenotypic expression of traits targeted by specific markers, leading to phenotypic variation and instability in marker effects. GxE interaction further complicate this dynamic, as specific genetic variations may express differently across various environments, causing the same marker to have varying effects depending on environmental conditions. For example, an FM associated with drought tolerance may be effective under drought conditions but less so under combined stress factors or other environmental contexts. Due to these environmental influences, markers validated in one environment may not guarantee the same results in other settings. Consequently, breeding programs using FMs must rigorously test marker stability and effectiveness across diverse environmental conditions (Xu et al., 2012; Salgotra and Neal Stewart, 2020). Transferability, another critical factor, involves the ability to apply FMs identified in one population or environment to another. This is particularly important for global breeding programs aiming to develop cultivars suited to diverse environments. To address these challenges, additional criteria beyond genetic linkage should be considered when defining a polymorphism as “functional.” These include the stability of marker effects across diverse contexts and consistent predictability of the target trait (Sargent et al., 2007). Thorough validation across multiple populations and environments is essential to confirm their reliability and ensure their intended roles in breeding programs. While FMs hold significant potential for improving plant breeding efficiency, their effectiveness depends on careful application and ongoing research to refine their use and address these limitations.

### 5.2 Future prospects for FMs

Over the next 20 years, FMs are expected to undergo significant advancements, driven by innovations in gene-editing, sequencing technologies, and multi-omics approaches, as well as increasing integration with artificial intelligence (AI) and machine learning (ML). One of the most transformative areas will be the creation of FMs with gene-editing technologies (Arora and Narula, 2017) by directly introducing specific mutations into genes of interest. Furthermore, as NGS costs continue to decrease, breeders will increasingly use whole-genome sequencing routinely, leading to the discovery of high-resolution polymorphisms in both coding and non-coding regions of the genome. This will include markers for regulatory elements, such as enhancers and promoters, which play important roles in gene expression (Salgotra et al., 2014). Looking forward, the application of ML-based gene–trait association models is expected to further enhance the predictive power of forward genetic approaches. By integrating vast datasets from genomic, phenotypic, and environmental sources, these models can identify complex and subtle gene–trait relationships, including non-linear interactions, that might otherwise be overlooked. This will ultimately accelerate genetic gain and facilitate the development of more resilient crop varieties (Negus et al., 2024).

The use of AI and ML will revolutionize the discovery and utilization of FMs and will have a profound impact on future breeding programs. In the phenotyping stage for FM development, large-scale visual data on target traits such as growth status, chlorophyll content, biomass, and disease resistance can be captured using various high-resolution imaging platforms including drones, satellites, ground-based sensers, and robots (Gill et al., 2022; Pinto et al., 2023; DeBruin et al., 2025). Image-based phenotyping enables accurate and consistent trait evaluation across large populations, offering significant efficiency over traditional manual scoring methods that can significantly reduce labor. At the core of this process is the convolutional neural network (CNN), a deep learning architecture specialized for image recognition and feature extraction (Fukushima, 1980). CNNs automatically learn to detect complex visual patterns and are widely used for tasks such as disease diagnosis, classification of healthy versus stressed plants, flowering time prediction, and automated leaf area estimation (Jiang and Li, 2020). Advanced CNN variants (e.g., ResNet, U-Net) also facilitate temporal image analysis, enabling dynamic monitoring of crop development and high-resolution extraction of phenotypic states at specific time points (Tausen et al., 2020; Malagol et al., 2025). These AI-driven phenotypic data can then be integrated with genomic information to support QTP discovery and FM development.

Recent advances in AI, ML and multi-omics technologies are transforming how causal variants are detected and have strong potential for FM development. ML algorithms are increasingly applied to large-scale genomic data to distinguish causal polymorphisms from background noise (Farooq et al., 2024). For example, models such as eXtreme Gradient Boosting (XGBoost) or deep learning (DL)-based tools like DeepVariant can effectively prioritize candidate SNPs that are likely to affect gene function or regulation, streamlining downstream validation (Poplin et al., 2018; Wang et al., 2025a). These tools improve the accuracy of variant calling by reducing false positives and enhancing the detection of rare or structurally complex polymorphisms that conventional methods often miss (Chen et al., 2023b). In addition, AI-assisted primer design platforms, some of which incorporate ML layers into existing tools help researchers select optimal primers for experimental validation, thereby improving amplification success and efficiency (Dwivedi-Yu et al., 2023; Ghorbani et al., 2025).

Integration of multi-omics data, such as transcriptomics, epigenomics, and chromatin accessibility, provides a more comprehensive view of how genetic variants influence gene expression and phenotypes. By correlating sequence variants with expression profiles, regulatory modifications, and chromatin state across diverse tissues or developmental stages, researchers can prioritize variants that are more likely to be functionally relevant (Mahmood et al., 2022; Liu et al., 2025). Similarly, single-cell genomics is expected to advance, facilitating the identification of FMs (Cuperus, 2022). Single-cell genomics offers unprecedented resolution in understanding how individual cells respond to environmental stimuli or regulate complex traits. Unlike bulk sequencing, which averages signals across heterogeneous tissues, single-cell genomics enables the dissection of cell-type-specific gene expression and regulatory mechanisms. In plants, this approach has been successfully applied to uncover developmental trajectories in Arabidopsis root cells (Denyer et al., 2019) and to map stress-responsive transcriptional programs in rice and maize (Li et al., 2024; Wang et al., 2025b). These studies provide valuable insights into how specific cell types contribute to key agronomic traits such as drought tolerance, nutrient uptake, and disease resistance. Incorporating single-cell data into FM development enables the identification of variants that function in a cell-specific manner, thereby improving the precision and biological relevance of marker selection. This integrative approach enhances the resolution of causal variant detection and increases the biological confidence of selected FMs, ultimately improving the precision of marker development and downstream breeding applications. More importantly, this approach offers a more efficient strategy for FM development, significantly accelerating the overall process of FM development. As such, the convergence of AI, multi-omics, and advanced genomics is poised to become a cornerstone of precision breeding in the coming decades.

In response to environmental challenges, future breeding programs will focus on developing resilient crops using FMs for traits such as abiotic stress tolerance. FMs for genes controlling root architecture, water-use efficiency, and photosynthetic efficiency will become essential for developing varieties that can thrive under increasingly harsh conditions. Additionally, polygenic trait selection will become more refined through the use of multiple FMs, improving the ability to select for complex traits that enhance crop adaptation to different environments (Marsh et al., 2021). The next 20 years will also see the integration of FMs with multi-omics approaches, combining genomics with transcriptomics, metabolomics, and epigenomics. This view of gene-trait relationships will allow breeders to develop more precise FMs that reflect not only genetic variation but also its effects on gene expression and metabolic pathways (Cuperus, 2022).

Finally, due to the importance of global food security, international collaboration in plant breeding programs will become increasingly important. FMs will serve as a key tool for sharing genetic information across borders, ensuring that breeding efforts are aligned to address diverse environmental and agricultural challenges. Public genomic databases will play a significant role in facilitating this collaboration. These platforms will enable researchers and breeders from different areas to access and contribute to a shared pool of genetic data, including FMs and genomic variants. This will accelerate the discovery of new markers and their application in breeding programs, fostering the development of crop varieties that can thrive in various conditions while contributing to sustainable agricultural systems worldwide.
